# Grouping of chemicals for safety assessment: the importance of toxicokinetic properties of salicylate esters

**DOI:** 10.1007/s00204-024-03935-8

**Published:** 2025-01-04

**Authors:** Abdulkarim Najjar, Sébastien Grégoire, Beate Nicol, Andreas Natsch, Nazanin Golbamaki, Fanny Boisleve, Amaia Irizar, Brian Wall, Angus Swinscoe, Valérie Masini-Etévé, Kaushal Joshi, Anne Marie Api, Peter Griem, Allison Reis, Nicola J. Hewitt, Estefania Cardamone

**Affiliations:** 1 Beiersdorf AG, Unnastrasse 48, 20245 Hamburg, Germany; 2 L’Oreal Research & Innovation, Aulnay-Sous-Bois, France; 3 Unilever U.K, Colworth Science Park, Sharnbrook, UK; 4 Givaudan Schweiz AG, CH-8600 Duebendorf, Switzerland; 5 Chanel, Neuilly, France; 6 The International Fragrance Association (IFRA), Geneva, Switzerland; 7 Colgate-Palmolive Company, Piscataway, NJ 08854 USA; 8 The Estée Lauder Companies, Whitman Laboratories, Petersfield, UK; 9 Research Institute for Fragrance Materials (RIFM), Inc, Mahwah, NJ USA; 10 Symrise AG, Holzminden, Germany; 11 The Procter & Gamble Company, Mason, OH 45040 USA; 12 Scientific Writing Services, Erzhausen, Germany; 13 Cosmetics Europe, Brussels, Belgium

**Keywords:** Grouping, Risk assessment, Esters of salicylic acid, Metabolism factor, Skin absorption, Internal exposure

## Abstract

Grouping of chemicals has been proposed as a strategy to speed up the screening and identification of potential substances of concern among the broad chemical universe under REACH. Such grouping is usually based on shared structural features and should only be used for the prioritization objectives. However, additional considerations (as well as structural similarity) are needed, e.g., mode of action, metabolic pathways, chemical reaction products and physicochemical properties, when regulatory management measures are considered (such as restriction, harmonized classification and labeling). Guidance documents from the European Chemicals Agency (ECHA) recommend considering toxicokinetic information to enhance the robustness of the grouping; however, examples of this approach are lacking. Therefore, this paper shares findings on chemical grouping based on ADME data generated for multiple esters of salicylic acid. These differ with respect to chain length and branching of the alcohol moiety of salicylic acid ester, resulting in a wide range of lipophilicity (LogP 0.21–10.88). Since LogP impacts skin absorption, as well as hydrolysis by carboxylesterases, the bioavailability and thus internal exposure to topically applied salicylate esters can vary considerably. Therefore, we collected skin absorption and metabolism data for 41 salicylates using in vitro testing and in silico models and combined the information to group them according to their potential systemic exposure to the major metabolite, salicylic acid. The results show that, despite a similar general chemical structure, their toxicokinetics vary considerably, indicating the need for better understanding of ADME properties to assess the internal exposure for sound risk assessment.

**Supplementary Information:**

The online version contains supplementary material available at 10.1007/s00204-024-03935-8.

## Introduction

The Chemicals Strategy for Sustainability (CSS) is part of the European Union (EU) “Green Deal” to achieve zero pollution by banning harmful substances from consumer products. In 2020, the CSS proposed grouping as a regulatory strategy within the registration, evaluation, authorization, and restriction of chemicals in the EU (REACH), so that the evaluation of structurally similar substances is addressed as a group, rather than assessing the hazard of each substance individually (EC 2020). This approach has been progressively introduced through common screening, and since 2017, groups of substances of potential concern have been the main starting point for authorities’ work. The first ECHA report on Integrated Regulatory Strategy (IRS), published in April 2019, described how this concept could speed up the screening and prioritization of substances (of concern), whereby a substance that has not been assigned can be given a high priority for risk management, high priority for data generation or a low priority for further regulatory action. Thus, ECHA has moved from a substance-by-substance approach to the grouping of structurally similar substance approach.

The IRS report indicated that grouping is done primarily using IT-based algorithms and following two complementary methods, the first being structural similarity and read-across and the second being based on categories in registration dossier. The aim to group substances and assess them based on toxicological information on one or more structurally similar substances and/or common metabolites within the group i.e., using read-across, may be an effective way of filling data gaps for any safety assessment. In this strategy, relevant information from analogues (“source substances”) is used to predict the toxicological properties of “target” substances. This could reduce the need for in vivo experimental testing of every target substance. The IRS aimed to identify substances that need regulatory action, as well as those that do not. The CSS was reported by the European Chemicals Agency (ECHA) to have acted as a driver that has sped up the work considerably, from ~ 200 substance dossiers screened per year to ~ 1900 (ECHA 2022).

ECHA has been publishing multiple documents since 2021 denoted “Assessment of Regulatory Needs” (ARN), which are based on very loose structural properties and aim to understand and enhance pre-screening hazard assessment. ECHA proposes different levels of regulatory measures, including potential bans of substances based on chemical grouping defined only by common structural features. While this has enabled the identification of substances needing follow-up data generation, it has led to some pitfalls in the strategy and justified criticism specifically when risk management are concerned. This became especially evident when selected similarity criteria were insufficient in predicting toxicity (Wohlleben et al. 2023). Although structural similarity can be an obvious starting point for grouping, the approach also needs to consider the mode of action (MoA), toxicodynamic properties and toxicokinetics to increase the confidence in the assessment of similarity of the grouped substances. For example, if the toxic effect of the reference chemical arises due to saturation of the primary metabolic pathway, leading to a shift towards a toxic pathway, and the target chemical does not exhibit similar toxicokinetic properties, with no saturation of the primary pathway, it is scientifically unjustified to assume similar toxicity between the two chemicals. Consequently, relying solely on chemical structure for hazard classification may result in over-classification that does not reflect actual toxicological risks. The 2008 ECHA guidance recommends a stepwise approach to develop a grouping based on metabolic pathways, since toxicokinetic information can considerably strengthen the robustness of the read-across (ECHA 2008). Grouping and read-across for regulatory purposes should follow the ECHA guidance (ECHA 2008) and the Read-Across Assessment Framework (ECHA 2017). This has historically been perceived to establish considerable hurdles for REACH registrants when using grouping and data gap filling in their dossier submissions. Moreover, the guidance in these ECHA documents does not seem to be followed in ECHA’s ARN documents. This has led to a situation whereby the different benchmarks for groupings are used by regulators and dossier submitters, resulting in recent scientific discussions (Natsch et al. 2023).

This paper provides an example of a grouping not solely based on chemical similarity but also considering physicochemical and ADME (absorption, distribution, metabolism and excretion) properties. The project was initiated to share findings and recommendations on chemical grouping based on data generated for multiple esters of salicylic acid (referred to hereon as “salicylates”). The project was initiated as a result of the European Commission issuing a call to identify salicylates used in cosmetic products that are metabolized in humans to salicylic acid (EC 2022). The call was prompted by the two opinions from the Scientific Committee on Consumer Safety (SCCS) on the safety of homosalate (SCCS 2020) and methyl salicylate (SCCS 2021), in which concerns were raised over the potential additional and cumulative systemic exposure of consumers to salicylic acid from various salicylates used in cosmetic products. Additionally, ECHA published in 2021 an ARN for salicylate esters (ECHA 2021).

Salicylates are conjugates of salicylic acid, whereby the carboxylic acid group is esterified with an aliphatic or aromatic alcohol. Hydrophobicity increases with increasing aliphatic chain length on the alcohol part of the molecule, and since LogP is known to be a critical property for the absorption of chemicals through skin (Bunge and Cleek 1995; Grégoire et al. 2009b; Potts and Guy 1992), it can be expected that the bioavailability of topically applied salicylate esters can vary considerably. Dermal bioavailability will impact the internal exposure of substances and thus their potential to cause systemic toxicity. In vitro data on skin absorption were available for some but not all salicylates included in our study. Since the generation of in vitro skin absorption data is resource—and time—consuming, the use of in silico predictions can provide a fit-for-purpose conservative estimate (Grégoire et al. 2021). In silico models may also be the only way of estimating the systemic exposure of some chemicals which are topically applied due to technical difficulties in conducting the in vitro skin absorption assays, e.g. lack of a radiolabel and poor analytical sensitivity, non-specific binding, insolubility etc. Here, we used two in silico models to predict the skin absorption of salicylates present in a typical cosmetic formulation, which we refer to as the “Local” and “Global” models. The Local model was developed based on a small set of salicylates only (described in the Methods section) and the Global model, as the name suggests, is a published model built using a large set of data covering different chemical classes and structures (described in detail by Gregoire et al. (Grégoire et al. 2009a, b)). During the project additional in vitro skin absorption data were generated for six substances to further strengthen the evidence.

Once entering the systemic circulation, esters can be hydrolyzed in vivo by carboxylesterases present in the liver (Ross et al. 2012). However, not all salicylates release the same amount of salicylic acid over a given time frame since they are likely to have different affinities for the carboxylesterases involved in their hydrolysis (Najjar et al. 2024). Therefore, salicylates cannot be regarded as equal in terms of their contribution to systemic concentrations of salicylic acid. This was demonstrated by Najjar et al. (2024), who showed that salicylates with lower LogP values generally exhibited higher in vitro clearance values in incubations with liver S9 fractions. This trend in intrinsic clearance correlated with the substrate specificity of the main liver carboxylesterase enzyme, carboxylesterase-1 (Fagerberg et al. 2014; Ross et al. 2012), which preferentially hydrolyses esters with a large, bulky acyl group and a small alcohol group. Therefore, a so-called “Metabolism Factor” (MF) to account for the differences in the release of salicylic acid was derived using a High Throughput Pharmacokinetic (HTPK) Model to estimate the percentage converted to salicylic acid in humans in 24 h (Najjar et al. 2024). The HTPK Model converts the intrinsic clearance values (CL_int_) to in vivo clearance and half-life values and is a suitable tool for providing robust values in a relatively short time (Liu et al. 2020; Naga et al. 2022).

We collected physicochemical properties, skin absorption, metabolism, and MF data for 41 salicylates using in vitro and in silico models and combined the information to group them according to their potential to contribute to internal exposure to salicylic acid. The results were used to illustrate how grouping using toxicokinetics can help to differentiate the level of toxicological concern between substances identified as having a similar hazard solely based on chemical similarity.

## Materials and methods

### Chemical selection

**Fig. 1:**
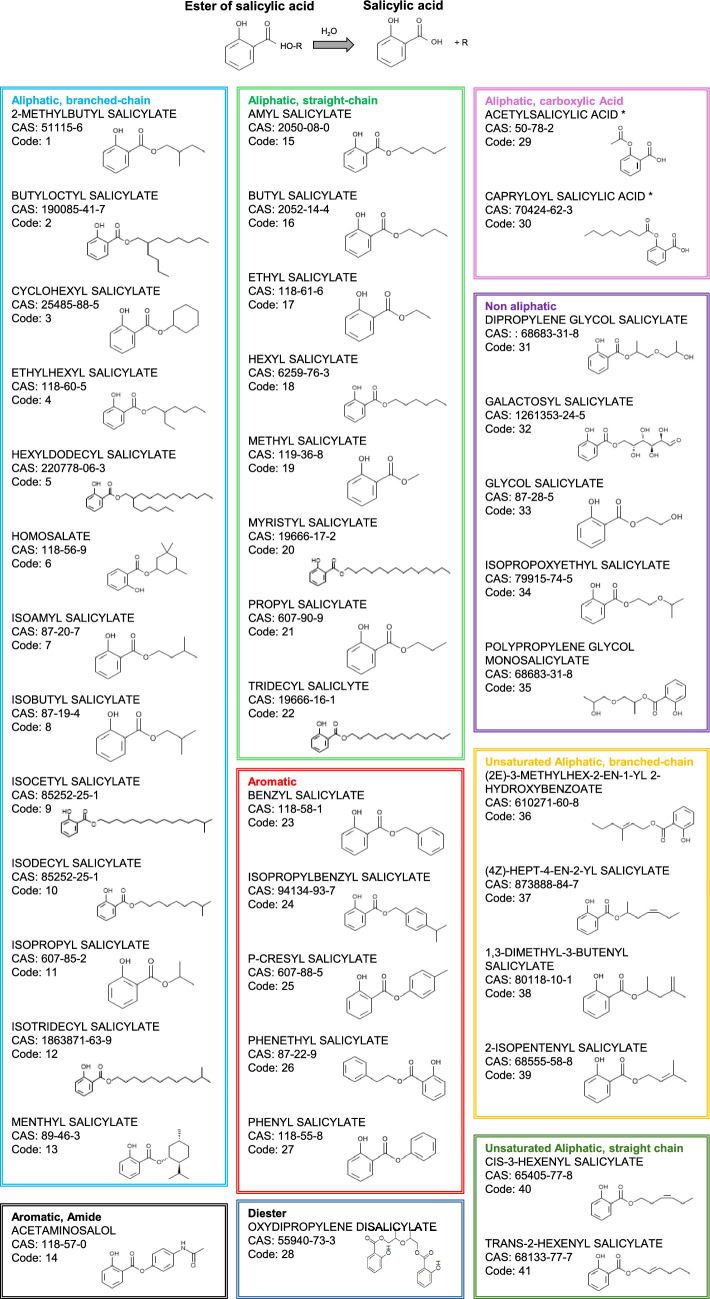
Structure and hydrolysis of esters of salicylic acid. The code number and color relate to the identity of the salicylates used in Figs 7 and 8. The generic structure at the top of the figure does not include acetylsalicylic acid or capryloyl salicylic acid (denoted with an asterisk)

The method of chemical selection is described by Najjar et al. (2024). Forty-one salicylates were selected (Fig. 1). These were restricted to single substances (as opposed to mixtures), which were classified as salicylates according to the structure shown in Fig. 1. The exceptions to this are acetylsalicylic acid and capryloyl salicylic acid, which are aliphatic carboxylic acid structures (denoted in the figure with an asterisk). The common metabolic pathway for all these substances is hydrolysis to salicylic acid, catalyzed by carboxylesterases.

### Skin absorption data

Measured in vitro data were available for 15 substances (see Supplementary Table 1), the values of which were used in the chemical grouping. Of these, data were available from the literature for 9 substances and the remaining values for 6 substances were generated as described below and the summary of the results are shown in Supplementary Table 2). These studies were all compliant with the Organization for Economic Cooperation and Development (OECD) Test Guidelines (OECD 2004b) and SCCS recommendations (SCCS 2010, 2023). Only data for substances applied in typical cosmetic formulations with an exposure duration of 24 h were considered (thus, data for substances applied in solvents such as acetone or studies with a shorter exposure were excluded from the current evaluation).

For the 26 substances without measured data, predicted skin absorption values were derived using the Local and Global in silico models (described below).

#### In vitro skin penetration studies

The in vitro skin penetration of six chemicals applied in a typical body lotion (representative as a typical oil-in-water formulation used for many products) was tested in frozen human skin according to Good Laboratory Practice and according to the OECD Test Guideline 428 (OECD 2004b) and OECD Guidance Document No. 28 (OECD 2004a) and following the SCCS Basic criteria (SCCS 2010).

Unlabeled chemicals were acquired from Takasago International Chemicals (Europe) S.A., El Palmar Murcia, Spain (isopropoxyethyl salicylate), Sigma-Aldrich (glycol salicylate), Givaudan Suisse AS, Vermier, Switzerland (cyclohexyl salicylate and (4Z)-hept-4-en-2-yl salicylate) and from Hallstar (butyloctyl salicylate). Isotridecyl salicylate was tested using radiolabeled substance only. The radiolabeled chemicals were from Eurofins Selcia Ltd. (Ongar, UK). The body lotion formulation is a typical oil-in-water but proprietary base formulation from Beiersdorf AG, Hamburg, Germany. The radioactivity content and homogeneity of the dose preparation was confirmed by analyzing sub-samples of solvent dilutions by liquid scintillation counting. The radiochemical purity of > 99% and stability over 24 h was confirmed by HPLC-Flow Scintillation Analysis. All substances were confirmed to be sufficiently soluble in the receptor fluid (phosphate buffered saline supplemented with 6% (w/v) polyoxyethylene (20) oleyl ether (Brij O20)) to achieve sink conditions.

The volatility of the substances (shown in Supplementary Table 2) was measured by applying a dose of final formulation (2 or 10 mg/cm^2^) on 12 glass slides which were placed into Franz cells. Subsequently, 3 cells were left unoccluded, 3 cells had one carbon filter, 3 cells had 3 carbon filters and 3 cells were fully occluded using parafilm. The cells were maintained at 32 °C for 24 h, after which slides, donor cells and film were washed with acetonitrile and the wash analyzed by liquid scintillation counting. Carbon filters were extracted with acetonitrile and the extract analyzed by liquid scintillation counting.

Discs of human dermatomed skin (thickness of 400 µm) from 4 donors (*n* = 3 per donor) were thawed and mounted on static Franz diffusion cells. Skin integrity was assessed according to electrical resistance across the sample and only skin discs with a resistance of > 5 kΩ were used (Brackin et al. 2024). The skin was unoccluded and maintained at 32 ± 1 °C. The exposure area was 2.54 cm^2^. The receptor chamber contained 500 µL receptor fluid, which was stirred using a magnetic stirrer.

An amount of 2 mg/cm^2^ formulation containing test substance at a concentration of 0.5% (cyclohexyl salicylate, (4Z)-hept-4-en-2-yl salicylate, isopropoxyethyl salicylate), 1% (butyloctyl salicylate, glycol salicylate) or 0.1% (isotridecyl salicylate) was applied to the skin surface. The concentrations of the substances were selected considering the following: In defense regulatory dossiers, the ingredient is usually tested at its maximum use concentration; however, there is a risk that the substance does not fully solubilize at this concentration in the considered testing formulation (vehicle), which would artificially decrease the calculated % absorption. When the substance is fully soluble in the vehicle, the % absorption is assumed to be concentration-independent. Therefore, to be able to extrapolate the % absorption to different use concentrations, a lower and soluble concentration was selected. The amount of formulation applied (2 mg/cm^2^) exceeded the intended consumer use condition (1 mg/cm^2^) used here to achieve an even distribution over the skin disk.

Receptor fluid samples were taken just prior to treatment and at 1, 2, 3, 4, 6, 8, 10, 12, 16, 20 and 24 h post application. The volume of fluid in the receptor chamber removed was immediately replaced by an equal volume of fresh receptor fluid. After 24 h, the skin was washed three times with a natural sponge pre-wetted with 3% Teepol L® in water. The sponges were digested in Goldisol® and made up to a recorded volume before analysis. The receptor chamber was washed with acetonitrile and the sample was analyzed by liquid scintillation counting.

The stratum corneum (SC) was removed by tape stripping using Scotch 3 M Magic Tape, to a maximum of 20 strips. Tape strips 1–5 were extracted individually, while the remaining 15 tape strips were combined in groups of five (6–10, 11–15 and 16–20) prior to overnight extraction in acetonitrile. The epidermis on the remaining skin disc was separated from the dermis using a heat separation technique (Hewitt et al. 2020). The epidermis and dermis were separately digested in Goldisol® and a sample of the digests was analyzed by liquid scintillation counting.

The bioavailable amount was calculated as the % of the applied dose recovered in the epidermis, dermis and receptor fluid.

#### In silico skin absorption models

The Global and Local models were used to predict the % absorption of substances applied in a body lotion formulation over 24 h (results are listed in Supplementary Table 1).

##### Global model

The Global model was developed in four steps: (1) prediction of skin absorption (cumulative mass over 24 h of a chemical absorbed into and across the skin (i.e., total skin + receptor fluid) from an aqueous vehicle, (2) correction for cosmetic vehicles, (3) correction for the amount in the SC, and (4) inclusion of chemical volatility. It was built on the following assumptions. First, despite application of a finite dose of formulation, a steady state can be achieved. This assumption is supported by the data showing that for a wide range of chemical and cosmetic vehicles, most of the chemical remains at the skin surface (Haque et al. 2016; Tampucci et al. 2018). Moreover, it is a conservative assumption as the flux for an infinite dose is always greater than the flux for a finite dose at the steady state. All cosmetic formulations used were treated as oil-in-water emulsions. It was assumed that only the fraction of chemical in the aqueous phase was able to partition with the SC.

*Step 1: Starting equations for water as the vehicle*

The equations (Eqs. 1 and 2) proposed by Cleek and Bunge (Bunge et al. 1995) to calculate the amount of chemical leaving the vehicle and entering the skin were used. Considering ${t}^{*}$, the time to reach steady state,

for $${t}_{exp}\le {t}^{*}$$

1 $$\frac{{Q_{{{\text{Total}}{\kern 1pt} {\kern 1pt} {\text{Skin}}{\kern 1pt} { + }{\kern 1pt} {\text{RF}}}} }}{{AC_{v}^{o} }} = 2K_{sc/w} \sqrt {\frac{{D_{sc} t_{\exp } }}{\pi }}$$

for $${t}_{exp}> {t}^{*}$$

2 $$\frac{{Q_{{{\text{Total}}{\kern 1pt} {\kern 1pt} {\text{Skin}}{\kern 1pt} { + }{\kern 1pt} {\text{RF}}}} }}{{AC_{v}^{o} }} = \frac{{Kpt_{\exp } }}{1 + B} + K_{sc/w} h\frac{{1 + 3B + 3B^{2} }}{{3(1 + B)^{2} }}.$$

The Potts & Guy relationship (Potts and Guy 1992) (Eq. 3) was used to calculate the permeability coefficient (Kp) and the corresponding diffusion coefficient in the SC, ${D}_{sc}$, and chemical partition coefficient between SC and water, ${K}_{sc/w}$.

3 $$\log Kp \left( {cm/h} \right) = - 2.71 - 0.0061 MW + 0.71\log D_{{pH {\text{vehicle}}}}$$

where *t*_exp = time of exposure, ${Q}_{Total Skin+RF}$ = Cumulative amount of chemical that permeates the skin and in the Receptor fluid; $h$ = thickness of the SC set at 10 µm; $A$ = Exposure surface area; ${C^\circ }_{v}$ = the initial chemical concentration in the vehicle; ${K}_{sc/w}$ = chemical partition coefficient between SC and water (calculated as follows: $\text{log}{K}_{sc/w}= 0.71{\text{log}D}_{pH vehicle}$); ${D}_{sc}$ = chemical diffusion coefficient in the SC (calculated as follow: $\text{log}\frac{{D}_{sc}}{h} (cm/h) = -2.71-0.0061 MW$). The LogD is used instead of LogP to correct for the ionization state of the permeant (Grégoire et al. 2009a).

B defines the ratio of permeability between SC and viable epidermis (Cleek and Bunge 1993).

$$B = \frac{{Kp\sqrt {MW} }}{2.6}$$

For $$B\le 0.6$$

$$t^{*} = \frac{{0.4h^{2} }}{{D_{sc} }}$$

For $$B>0.6$$

$$t^{*} = \left( {b - \sqrt {b^{2} - c^{2} } } \right)\frac{{h^{2} }}{{D_{sc} }}$$

With $$b = \frac{2}{\pi }\left( {1 + B} \right)^{2} - c$$

$$c = \frac{{1 + 3B + 3B^{2} }}{{3\left( {1 + B} \right)^{2} }}$$

*Step 2: Adaptation for use with formulations—training set*

These equations only apply for water as the vehicle; however, cosmetic vehicles are designed to solubilize non-aqueous soluble chemicals at a concentration higher than the maximum solubility in water. Thus, these equations were corrected to consider non-aqueous vehicles. The ratio of permeability between the SC and viable epidermis is independent of the vehicle as the vehicle does not modify their properties (Bunge et al. 1995). Potential penetration enhancement effects of the vehicle were excluded based on data showing that this is not the case for typical cosmetics formulations (Grégoire et al. 2009a).

The amount leaving the vehicle and entering the skin (e.g. total skin + receptor fluid) (Eqs. 1 and 2) was corrected accordingly, considering vehicle as an oil-in water emulsion (Eq. 4).

4 $$\frac{{C_{w} }}{{C_{v}^{0} }} = \frac{{f_{w} }}{{f_{w} + \left( {1 - f_{w} } \right) \times 10^{{a x log D_{pH vehicle} }} }}$$

where ${C}_{w}$= the concentration in the water phase of the model oil-in water emulsion

${C}_{v}^{0}$= the concentration of the chemical in the formulation

${f}_{w}$= Fraction of the substance that is water-soluble

*a* = A coefficient that may represent a scaling factor related to how LogD affects absorption

${\text{log}D}_{pH vehicle}$= the partition coefficient between octanol and water of the chemical at the pH of the vehicle

A training set of the model consisting of 101 individual data points corresponding to 36 chemicals tested in OECD TG 428 compliant ex vivo human skin studies was used to optimize parameters $a$ and ${f}_{w}$ in Eq. 4. Optimal performance for all formulations used in the training set was found with ${f}_{w}$=0.5 and $a=1-0.032 log {D}_{pH vehicle}$ (Grégoire et al. 2009a).

*Step 3: Correction for dermal delivery*

To calculate the dermal delivery, which only considers the amount in viable epidermis (VE) + dermis (D) + receptor fluid (RF), Eqs. 1 and 2 were corrected for the amount of chemical in the SC. The relative permeability of the SC to the VE is assumed to be independent of the vehicle since the vehicle does not modify the properties of VE and SC (Cleek and Bunge 1993) (Eq. 2). As VE is more hydrophilic than the SC, the resistance of the VE increases for lipophilic chemicals; hence, the permeability ratio, denoted as B in Eq. 5, increases with logP. Lipophilic chemicals do not easily enter the VE, leading to their accumulation in the SC.

5 $$B = \frac{{Kp_{SC} }}{{Kp_{VE} }}$$

The relative ratio of permeability is assumed to be proportional to the ratio between the SC and amount entering the VE and the absorbed amount (i.e. VE + D + RF) (Eq. 6).

6 $$B \cong \frac{{Q_{SC} }}{{Q_{VE + D + RF} }}$$

The amount in the Total Skin + RF is equal to the sum of absorbed amount and the amount in the SC:

7 $$Q_{Total Skin + RF} = Q_{SC} + Q_{VE + D + RF}$$

Combining Eqs. 6 and 7, the amount Total Skin + RF is corrected by a factor B to calculate the amount VE + D + RF (Eq. 8).

8 $$Q_{VE + D + RF} = Q_{Total Skin + RF} \frac{1}{1 + B}$$

*Step 4: Inclusion of chemical volatility*

The initial model was refined to include the impact of volatility as a rate limiting step for skin absorption (Grégoire et al. 2009a). This is important for three of the esters of salicylic acid which exhibit some significant volatility (ethyl salicylate, isopropyl salicylate and methyl salicylate, see Supplementary Table 2).

The evaporation rate k_evap_ (g.cm^−2^.h^−1^) was calculated using Eqs. 9, 10 and 11 (Kasting and Miller 2006).

9 $$k_{evap} = \frac{{k_{g} P_{VP} MW}}{{7.610^{5} RT}}$$

where *MW* (g.mol^−1^) is the molecular weight; *P*_*VP*_ (torr) is the vapor pressure; *R* (L.atm.mol^−1^.K^−1^) is the universal gas constant; *T* (*K*) is temperature (set at 305 K) and kg (cm.h^−1^) is the mass transfer coefficient in air calculated according to Eq. 10.

10 $$k_{g} = \frac{{6.320u^{0.78} }}{{MW^{1/3} }}$$

with *u* the air velocity (in m.s^−1^) adjacent to the skin (Stempfer and Bunge 2005).

Thus, the evaporated amount is calculated using Eq. 11.

11 $$Q_{evap} = t k_{evap}$$

*Input parameters and output values*

The Global model uses MW and LogD (defined at a given pH, calculated using the LogP and pKa), pKa and the vapor pressure (values for the substances evaluated here are shown in Annex Table 1). Since exposure time, amount of formulation and pH of the formulation are variable parameters, the following scenario was used to predict the values shown in Table 4: 2 mg/cm^2^ in a formulation with a pH of 6.5 applied for 24 h on ex vivo human skin. For volatile chemicals, the concentration of chemical in the formula was arbitrarily set at 1%. Only three chemicals are sufficiently volatile to limit their skin absorption (ethyl salicylate, isopropyl salicylate and methyl salicylate).

The final model predicts (in vitro) dermal delivery (i.e., the percentage in the epidermis, dermis and the receptor fluid of an infinite dose of test substance (Gregoire 2011)).

*Applicability domain of Global model*

MW and lipophilicity (LogD, calculated using the LogP and pKa) are parameters that describe the chemical applicability domain of the Global model. The validation set included 289 individual datapoints from 73 chemicals (including 6 salicylates) in different formulations. The MW of this dataset ranged from 76 to 741 g/mol and the logD from – 3.27 to 9.4, with most chemicals being within the MW range of 100 to 400 g/mol and LogD – 3.27 to 6. There are no measured in vitro skin absorption data for chemicals with higher MW or LogD values; therefore, the applicability domain was restricted to the second highest MW at 563 g/mol and second highest LogP at 6.2. If the substance does not fit with one of these criteria, the prediction quality is affected:

Extrapolation can be conducted for: MW > 541 and MW < 741 g.mol_−1_

Extrapolation can be conducted for: LogD > 6.3 and LogD < 9.3

Extrapolation can be conducted for: LogD > -5.3 and LogD < -3.3

Chemical having zwitterionic status at tested pH can lead to uncertainty on calculated LogD

If the MW is higher than 741 or the LogD higher than 9.3 and lower than − 5.3, no calculation is performed using this model and an assumption can be made that the level of skin absorption is lower than 1% of the applied dose.

*Validation of the Global model using a dataset containing multiple chemical classes*

This model was validated by Grégoire et al. (2009a) and was re-evaluated by us by expanding the number of data points to include a set of experimental 289 data points corresponding to 76 chemicals (of which 6 were salicylates). Inter-laboratory studies have shown in vitro experimental variation of about a factor 2 despite a standardized protocol (Wargniez et al. 2017). Thus, a difference between predicted and measured values lower than a factor 2 can be considered to be within the variability of the experimental data. For the majority of chemicals and formulations, the model tends to overestimate the skin absorption. Only a very small fraction of the data, 1.4% (4 data points) were underpredicted by more than a factor of 3. No data point was underpredicted by more than a factor of 4.

##### *Local model*

*Training set*

**Fig. 2:**
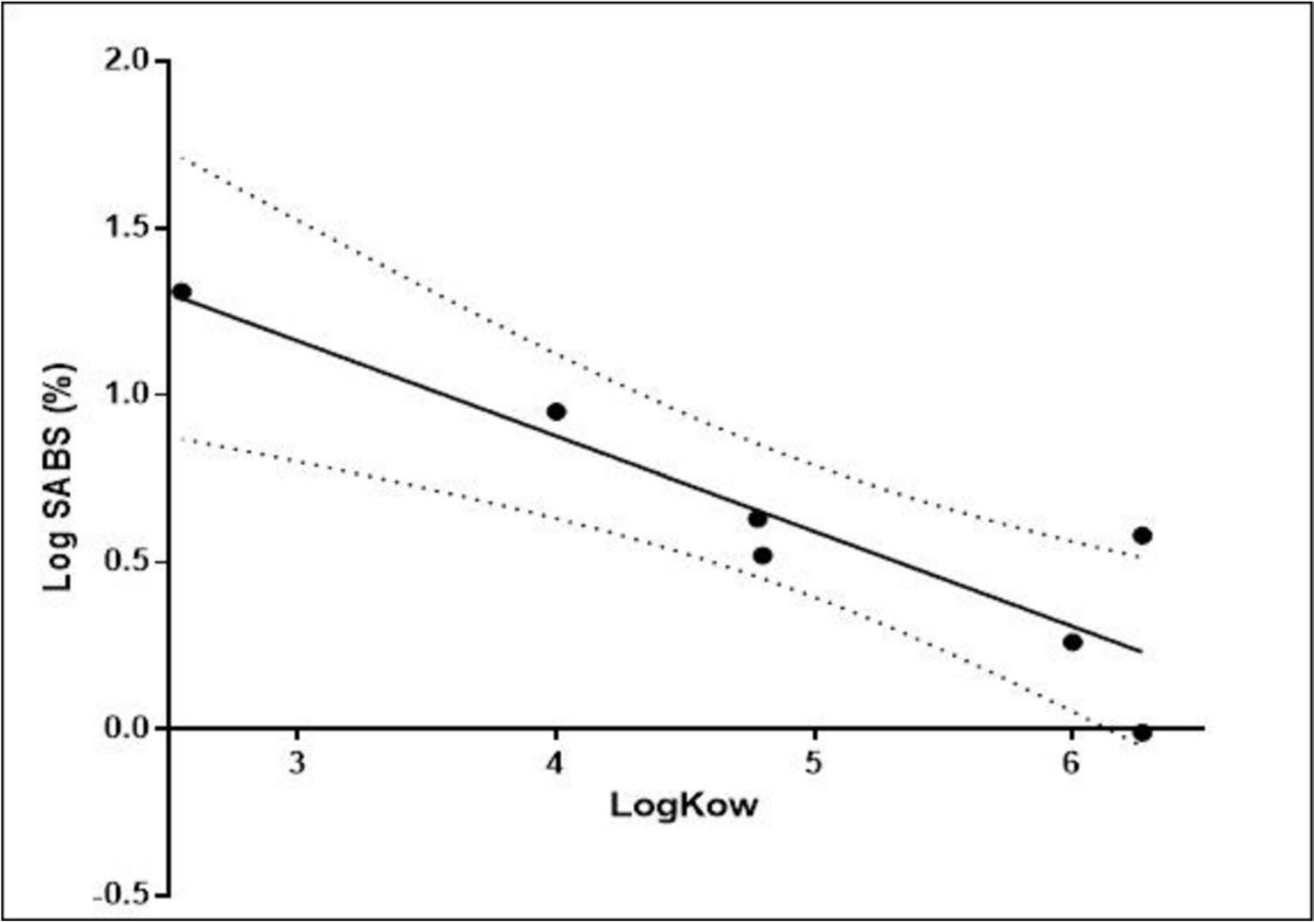
Correlation between skin absorption data and LogP of salicylates. (*n* = 7 data points)

The training set for the Local model included seven data points associated with six salicylates. There are two experimental values for homosalate which both comply with the SCCS guidance for in vitro skin absorption; therefore, both values were considered. This training dataset comprised values available at the start of the study (the remaining data for 9 substances were only available after the model had been developed). The experimental values for the % applied dose absorbed were: 9.01% for benzyl salicylate, 1.82% for ethylhexyl salicylate, 4.31% for isoamyl salicylate, 20.2% for methyl salicylate, 3.29% for phenethyl salicylate and 0.97% and 3.83% for homosalate (see Supplementary Table 1). The correlation between the LogP values for these substances and the % applied dose absorbed (Fig. 2, R^2^ = 0.817) resulted in Eq. 12, which was subsequently used for all substances. LogP was used for the correlation rather than the LogD since most of the salicylates are non-ionized (the exceptions being acetyl salicylic acid and capryloyl salicylic acid); therefore, the correction of ionization was not necessary.

12 $$Local \, Model = Log \left( {\% Applied \, dose \, absorbed} \right) = 2.016 - 0.2848 \times LogP$$

It is acknowledged that skin absorption is also a function of MW (Bunge and Cleek 1995); however, the correlation of absorption with MW was not as robust as that with LogP, since five of the six substances had MW values within a small range (208 to 262, listed in Supplementary Table 3). Therefore, to avoid over-fit the data and provide a simple model, we used only LogP as the input parameter.

*Applicability domain of the Local model*

The applicability domain was defined based on the LogP values of the substances used in the training set that ranged from 2.6 to 6.3. There were several substances which were outside the applicability domain of the Local model, with LogP values either lower than 2.6 or higher than 6.3. For substances with a LogP < 2.6, a default value of 50% was applied (according to the SCCS note of guidance (SCCS 2023)) and for substances with a LogP > 6.3, a conservative default value of 1.7% absorption was taken (i.e. the value obtained for a LogP of 6.3 using Eq. 1):

$$Log \left( {\% Applied \, dose \, absorbed} \right) = 2.016 - 0.2848 \times 6.3 = 0.222$$

$$\% {\text{ of the applied dose }} = { 1}0^{{0.{222}}} = { 1}.{7}\% .$$

*Uncertainty assessment of the Global and Local models*

The uncertainty of the models is related to the precision of the physicochemical parameters used, particularly LogP. Experimental precision of LogP is usually around 0.3 (according to the OECD 107 Guideline (OECD 1995)). Moreover, even in well conducted in vitro skin absorption studies it was considered that the variability could be equal to 100% (SD = mean). Thus, it was assumed that the model could theoretically not be more precise than a factor 2.

For both models, the same LogP values were used. These were measured values when available and predicted values from the EpiSuite software (EpiSuite Calc KOWIN v1.67) when they were not available. There were measured LogP data for 15 substances available. The correlation between measured LogP and LogP values calculated with EpiSuite is very good (r^2^ = 0.97, LogPexp = 0.083 + 0.988 log Kow) for these substances, indicating that predictions of skin absorption were not significantly impacted by the source of the LogP within this chemical space.

### Metabolism factor (MF) values

The MF values were taken from Najjar et al. (2024). These were generated using the High-Throughput PharmacoKinetics (HTPK) model from Simulations Plus (https://www.simulations-plus.com/), described by Liu et al. (2020) and (Naga et al. 2022). This software estimated the *t*_1/2_ in human plasma using input data including predicted values for plasma protein binding and either predicted intrinsic clearance (CL_int_) or measured CL_int_ calculated from data from incubations with human liver S9 (Najjar et al. 2024). The *t*_1/2_ in humans was used to calculate the MF i.e., % of the conversion of the substance in 24 h, according to Eq. 1.

13 $$Metabolism\,Factor \left( \% \right) = \left( {1 - 0.5^{{\left( {\frac{t}{{in vivo t_{1/2} }}} \right)}} } \right) \times 100$$

where *t* is the time passed since exposure of the chemical; 24 h (1 day) and in vivo *t*_1/2_ is the half-life of the substance in human plasma.

Measured values for F_up_ were not available for most of the substances; therefore, values were derived using in silico-only input and in silico F_up_ combined with measured CL_int, in vitro_ using human liver S9 (Najjar et al. 2024).

## Results

### Chemical mapping

**Fig. 3:**
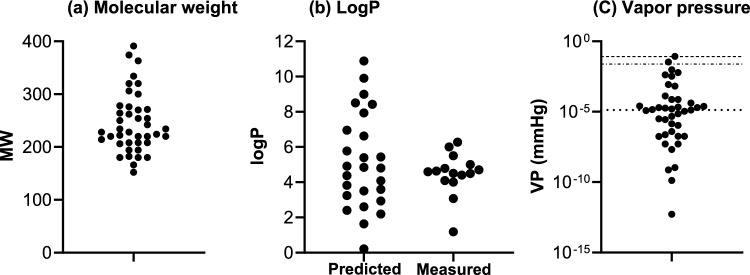
Ranges of physicochemical properties of the salicylates: **a** MW, **b** predicted and measured values of LogP and (**c**) volatility (vapor pressure). The dotted lines in (**c**) denote VP thresholds linked to the total recovery of a substance tested in a skin penetration study (Grégoire et al. 2019). VPs < 1.3 × 10^–5^ mm Hg = more likely to achieve > 90% total recovery; VPs between 1.3 × 10^–5^ and 2.4 × 10^–2^ mmHg = some volatility (< 30% loss), VPs > 8.2 × 10^–2^ mm Hg should be expected to evaporate to some extent during the course of a skin penetration study

The mapping of registered substances i.e., the “chemical space”, was conducted using three main physicochemical properties known to impact skin penetration, i.e., MW, LogP and volatility. These values were plotted to visualize the ranges of values (Fig. 3). The MW of the salicylates considered here ranged between 152 g/mol (methyl salicylate) and 391 g/mol (hexyldodecyl salicylate) (Fig. 3a). In addition to the saturation (which has toxicological impact), these salicylates differ mainly with respect to the chain length and branching of their alcohol moieties. Chain lengths ranged from one carbon atom in methyl salicylate up to 13 carbon atoms in tridecyl salicylate. The increase in chain length was concomitant with an increase in hydrophobicity—from a LogP of 2.60 for methyl salicylate up to a LogP of 8.99 for myristyl salicylate. The highest LogP was for the branched chain salicylate, hexyldodecyl salicylate (10.88) and the lowest was for galactosyl salicylate (for which the LogP was 0.21) (Fig. 3b).

The volatility of a topically applied substance will indirectly impact its bioavailability since the volatized fraction of a substance cannot enter the skin (Grégoire et al. 2019; Hewitt et al. 2020). Based on a correlation of the vapor pressure (VP) with the mass balance of chemicals tested in skin penetration studies (Grégoire et al. 2019), substances with a VP lower than 1.3 × 10^–5^ mmHg are more likely to achieve > 90% total recovery; substances with a VP between 1.3 × 10^–5^ and 2.4 × 10^–2^ mmHg could exhibit some volatility (< 30% loss), and substances with a VP greater than 8.2 × 10^–2^ mmHg should be expected to evaporate to a significant extent during the course of a skin penetration study. Of the substances considered, methyl and ethyl salicylate (with the highest VPs of 0.0343 and 0.0843 mmHg, respectively) were expected to be more volatile than others (Fig. 3c).

### Skin absorption values

**Fig. 4:**
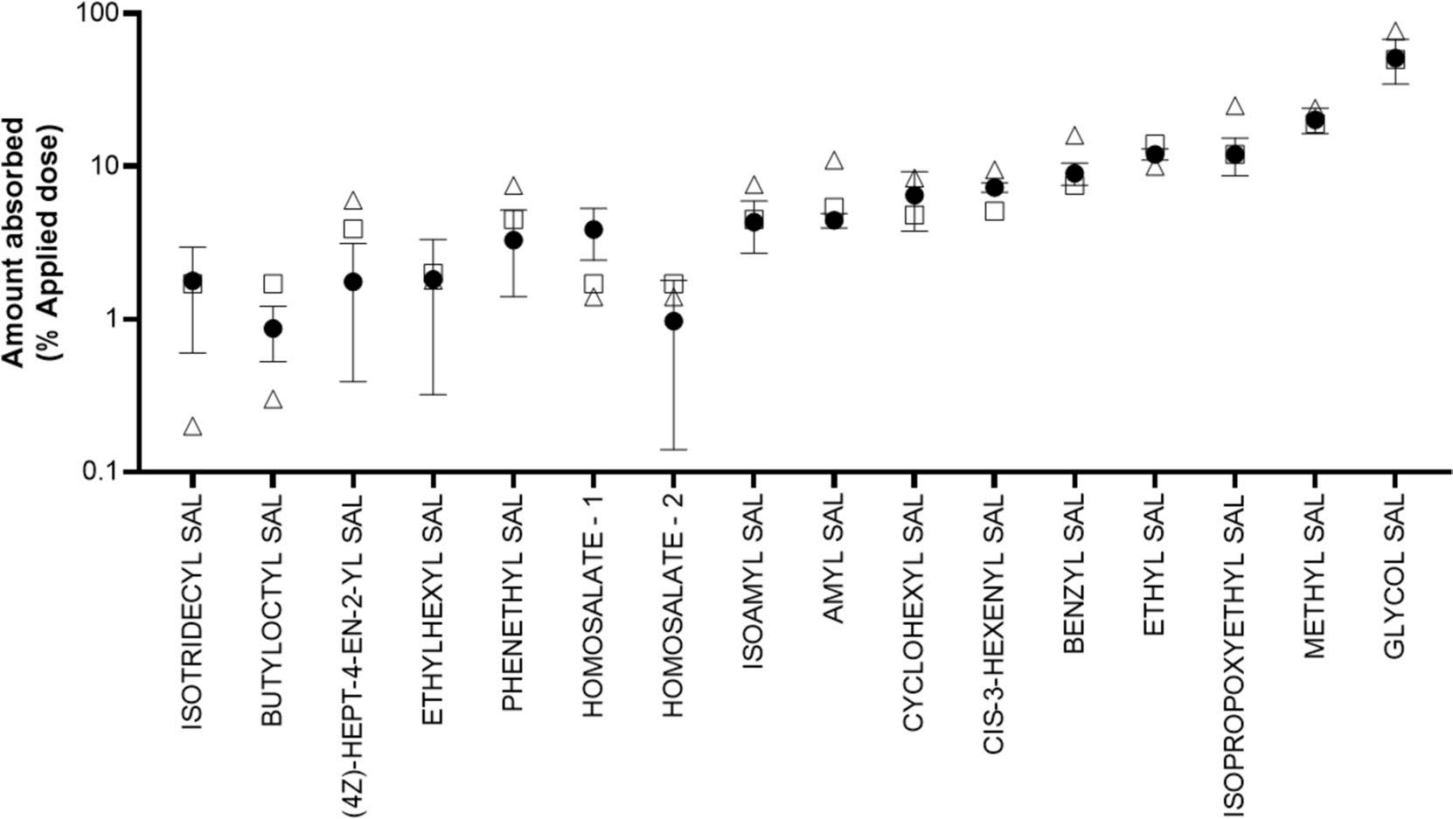
Comparison of measured skin absorption values (black circles) with values predicted using the Global (open triangles) and Local (open squares) models. Values for the measured absorption are a mean ± SD for 12 skin discs from 4 donors. Note that two measured values were available for homosalate. SAL = salicylate

Figure 4 shows that the measured skin absorption values for 15 salicylates were all well predicted by the Global and Local models. The predicted values were a mean of 1.28-fold (Global) and 0.94-fold (Local) of the mean + 1SD measured values. The goodness of fit according to the R^2^ was 0.95 for the Global model and 0.99 for the Local model (for all 15 substances and for the 10 substances not in the training set). For the grouping, in vitro data were taken even when the measured values were lower than the predicted values. For substances lacking measured values, a conservative approach was used, whereby the higher value from the Global and Local models was taken.

**Fig. 5:**
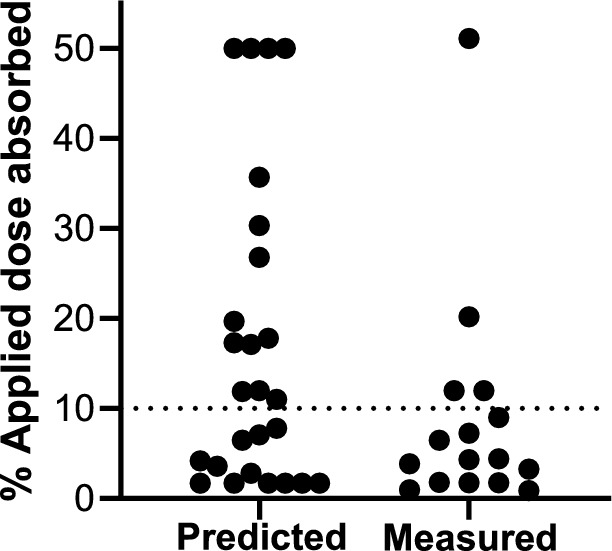
Ranges of skin absorption for the salicylates. The dotted line denotes skin absorption of 10% of the applied dose. The values for skin absorption are listed in Supplementary Table 1. Skin absorption values were predicted or measured in OECD and SCCS compliant experiments. Three/four values of 50% were default values since the LogP was out of the applicability domain for the Local model

There were three clusters of substances which were absorbed through skin to different extents. Over half of the substances (23 out of 40) had measured or predicted skin absorption values lower than 10% of the applied dose (Fig. 5). Thirteen substances had measured or predicted skin absorption values of between 11 and 36% of the applied dose, and only four substances had skin absorption values of 50% of the applied dose (three of which were default values for substances outside the applicability domain of the Local model (i.e. the LogP was < 2.6), according to the SCCS note of guidance (SCCS 2023) (see Sect. “Local model”)).

### Metabolism factor (MF) values

**Fig. 6:**
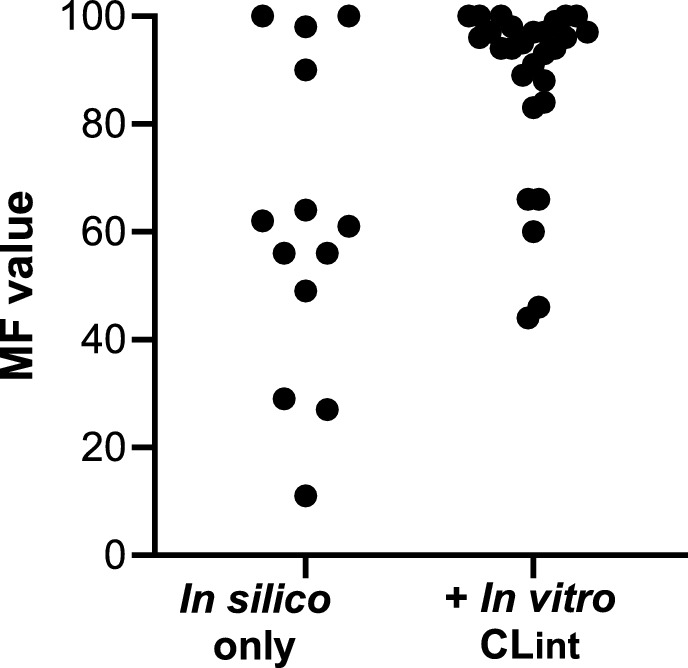
Ranges of MF values for the salicylates. The values for MFs are listed in Supplementary Table 3. MFs were estimated using either in silico only input parameters (“In silico only”) or using in vitro intrinsic clearance values from incubations with human liver S9 (“ + In vitro CLint”)

The MFs after “intravenous administration” (i.e., excluding the skin penetration and skin metabolism after topical application) calculated by (Najjar et al. 2024) are shown in Fig. 6. This revealed three clusters of substances which were hydrolyzed by the liver to different extents. There were 27 substances which were readily hydrolyzed within 24 h (> 80% metabolized in 24 h), 11 substances which were moderately hydrolyzed (by between 40 and 80% metabolized in 24 h) and three slowly hydrolyzed (11–29% metabolized in 24 h). The latter were oxydipropylene disalicylate, which a diester, 2E-3-methylhex-2-en-1-yl 2-hydroxybenzoate, which is a branched chain salicylate with a LogP value of 5.39, and trans-2-hexenyl salicylate, which is a straight-chain salicylate with a LogP of 4.84 (see Fig. 1 for structures).

### Chemical grouping

A comparison of the estimated internal exposure to salicylic acid after topical application of substances in a body lotion formulation was visualized by grouping substances according to their skin penetration and MF values. Each group represents a similar exposure to salicylic acid based on the two main criteria and ranges:

- Extent of skin penetration: 0–10%, 10–40% and > 40% of the applied dose absorbed
- MF: % of parent ester converted to salicylic acid: 0–40%, 40–80% and > 80% converted in 24 h

**Fig. 7:**
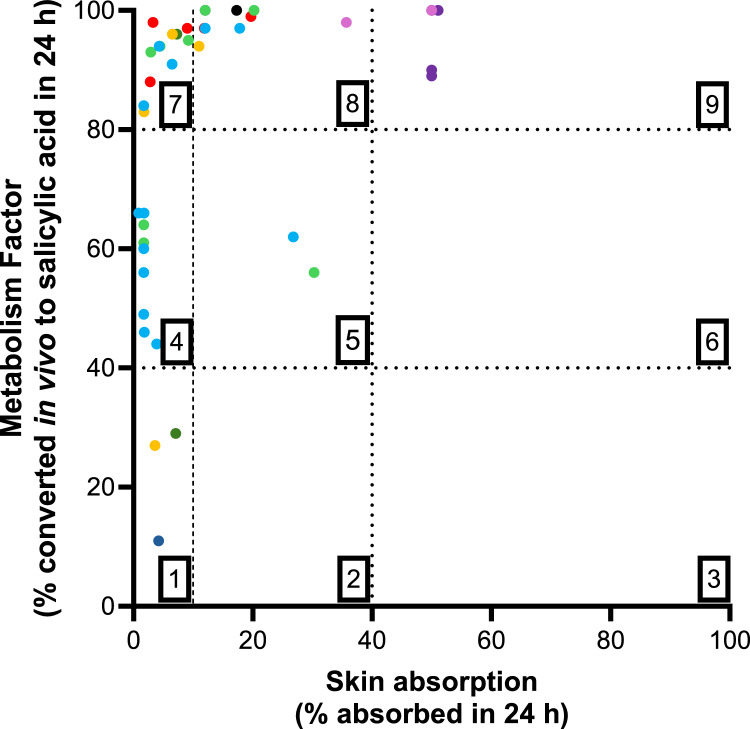
Substance grouping according to skin penetration and MF values. Skin absorption versus MF values for 41 salicylates. The numbers in each quadrant represent the group number. Substance color coding reflecting the different chemical/function group is listed in Fig. 1 and the grouping based on the skin absorption and MF values is summarized in Supplementary Table 4

These ranges are based on the three clusters of skin absorption and MF values visualized from Figs. 5 and 6. These ranges are mere suggestions and were set to show how these parameters could be used for Grouping– other ranges could also be used. When the current set of substances were clustered according to these criteria, there were five of the 40 salicylates in Group 9, representing the highest potential for systemic salicylic acid exposure (Fig. 7 and Supplementary Table 4). Most substances fell into Groups 7 and 8, indicating that exposure to salicylic acid was mainly driven by the extent of skin absorption rather than by the extent of hydrolysis. Fourteen of the substances with skin absorption values below 10% of the applied dose were more slowly metabolized (MFs were between 11 and 66%), indicating that the contribution to salicylic acid could be impacted by the extent of hydrolysis for some substances. Three of these substances, oxydipropylene disalicylate, ((2E)-3-methylhex-2-en-1-yl 2-hydroxybenzoate and trans-2-hexenyl salicylate), were allocated to Group 1, indicating they had the lowest exposure potential to salicylic acid.

## Discussion

Grouping of chemicals is increasingly proposed as a strategy to speed up chemical safety assessment. This approach can result in regulatory measures on an entire group of chemicals, typically based on shared structural features. Different levels of restrictions may be based on chemical groups defined only with respect to common structural features. However, additional considerations (in parallel with the structural similarity) may be needed, such as the MoA, metabolic pathways, chemical reaction products, different properties for isomers etc., to adequately reflect the real properties of substances and their toxicity profile (Yan et al. 2023). In particular, the role of metabolism in detoxification and toxification as well as toxicokinetic constraints should be considered. Guidance documents, such as from ECHA, already recommend considering toxicokinetic information to strengthen the robustness of the read-across justification; however, until now there has been a lack of published examples of this approach put forward for a large panel of substances. Therefore, this paper shares findings on chemical grouping based on ADME data generated for multiple esters of salicylic acid.

One of the challenges of assessing a large number of substances is that measured data may not be available or even possible to generate, e.g., due to a lack of availability of a radiolabeled form of the substance or analytical difficulties. Therefore, a pragmatic approach is to generate data for a sub-set of substances representing the chemical space of the substances being assessed. In this study, data for 15 substances tested in in vitro skin penetration studies were available. Measurements of skin absorption covered a broad range of lipophilicity, from a LogP of 1.63 (for glycol salicylate) to a LogP of 8.42 (for isotridecyl salicylate), thus representing the LogP range for most substances. The Global and Local in silico models were evaluated against the experimental sub-set and both correctly predicted the skin absorption over this LogP range which increased the confidence in their predictive performance in the investigated chemical space. Based on this thorough validation of the models with newly generated experimental data and their high predictivity, the values for substances without in vitro data were considered to be reliable.

The MFs for most substances (27 out of 41) were predicted using in vitro intrinsic clearance input data (Najjar et al. 2024). These were a good representation of the overall chemical space i.e. a LogP range from 1.63 (glycol salicylate) to 8.42 (isotridecyl salicylate), a MW range between 152 (methyl salicylate) and 391 (isotridecyl salicylate), and a VP range between 5.18 × 10^–13^ mmHg (galactosyl salicylate) and 0.0843 mmHg (ethyl salicylate).

The skin absorption and MF values were combined to separate the substances into nine groups. Theoretically, substances that are predicted to have a high level of skin penetration and are rapidly and extensively metabolized to salicylic acid (Group 9) may be more relevant to the overall systemic exposure to salicylic acid than those that are predicted to exhibit low skin penetration and form salicylic acid slowly (Group 1). The overall contribution to systemic salicylic acid exposure will also depend on their presence in cosmetics considering their concentration and frequency of use (which are not considered in this initial evaluation). Importantly, these results show that salicylates cannot be grouped based solely on structural similarity or the fact that they—in principle—produce a common metabolite of toxicological importance. While 27 of the substances were readily hydrolyzed (> 80%), only five were in the highest exposure group (Group 9). There were also substances which were poorly absorbed after topical application and not extensively hydrolyzed, namely oxydipropylene disalicylate, (2E)-3-methylhex-2-en-1-yl 2-hydroxybenzoate and trans-hexenyl salicylate, indicating that they would only result in low systemic concentrations of salicylic acid.

**Fig. 8:**
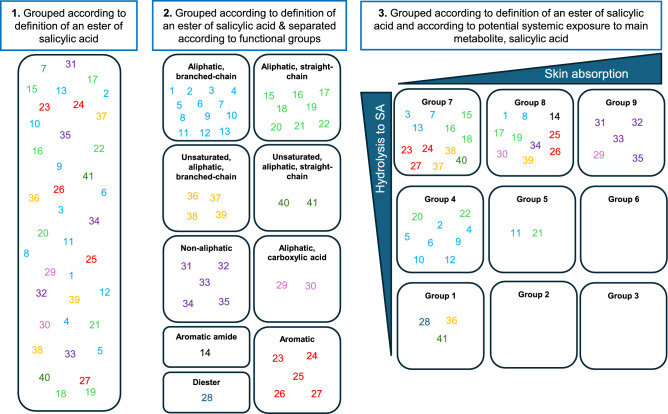
Grouping scenarios. Substances were grouped according to three definitions. Scenario 1: based on being an ester of salicylic acid only; Scenario 2: based on being an ester of salicylic acid and separated according to functional groups, and Scenario 3: based on being an ester of salicylic acid and separated according to potential systemic exposure to salicylic acid (skin absorption and hydrolysis to salicylic acid). Substance coding, skin absorption, MF values and Group numbers are summarized in Supplementary Table 4

A hypothetical example of the importance of sufficiently valid grouping is given in Fig. 8. This shows three scenarios whereby salicylates are grouped according to the definition: (1) of an ester of salicylic acid only; (2) of an ester of salicylic acid and separated according to their functional chemical groups, and (3) of an ester of salicylic acid and separated according to potential systemic exposure to salicylic acid (skin absorption and hydrolysis to salicylic acid). The latter relates to the concern of the regulatory call by the SCCS for aggregate systemic exposure to salicylic acid (EC 2022). Examples using several salicylates to show the impact of grouping according to their contribution to the systemic exposure to salicylic acid compared to grouping according to their chemical similarity are shown in Table 1. For the non-aliphatic substance, polypropylene glycol monosalicylate (in Group 9 and denoted as 35 in Fig. 8 and Table 1), the assumed overall exposure to salicylic acid of 50% of the applied dose based on the chemical similarity in the absence of ADME data is comparable with the exposure based on ADME Grouping, since it is in Group 9. However, when the estimated exposure of the unsaturated aliphatic substance, (2E)-3-methylhex-2-en-1-yl 2-hydroxybenzoate (Group 1 and denoted as 36 in Fig. 8 and Table 1) is considered, there is a substantial difference in the estimation of overall exposure to salicylic acid between the two scenarios. The overall exposure is assumed to be 50% of the applied dose when applying a default skin absorption value of 50% and 100% hydrolysis; however, according to the ADME Grouping, the overall exposure to salicylic acid is only 1% of the applied dose (skin absorption and hydrolysis were only 3.6% and 27%, respectively). This difference between the method of grouping is also observed for substance 6 (the UV filter, homosalate) and 13 (the fragrance ingredient, menthyl salicylate), whereby the overall exposure is assumed to be 50% of the applied dose when applying default values but less than 2% of the applied dose when applying ADME Grouping. The ADME grouping for substance 11 (isopropyl salicylate) is higher, with an exposure of 16.7% using ADME grouping but still lower than that when using default values. Figure 8 also shows that substances with certain functional groups cannot be linked to a specific ADME Group since there is a mix of each substance structure in most groups. This indicates that grouping based only on chemical similarity results in similar assumptions being applied to groups of substances with a large range of physicochemical properties and thus ADME properties. This can result in false conclusions e.g., the contribution of a substance to the aggregated internal exposure to salicylic acid.

**Table 1:** Example substances to show the impact of grouping according to their contribution to the systemic exposure to salicylic acid compared to grouping according to their chemical similarity. Substances 36, 6, 11, 13 and 35 are (2E)-3-methylhex-2-en-1-yl 2-hydroxybenzoate, homosalate, isopropyl salicylate, menthyl salicylate and polypropylene glycol monosalicylate, respectively.

Group number	Substance code	Chemical similarity only		Including bioavailability and internal exposure to salicylic acid		
		50% absorption × 100% hydrolysis	Overall systemic exposure to SA	% Absorption	% Hydrolysis	Contribution of each substance to systemic exposure to salicylic acid
1	36	50% × 100%	50%	3.6%	27%	= 3.6% × 27% = 1.0%
4	6	50% × 100%	50%	3.86	44%	= 3.86% × 44% = 1.7%
5	11	50% × 100%	50%	27%	62%	= 27% × 62% = 16.7%
7	13	50% × 100%	50%	1.7%	84%	= 1.7% × 84% = 1.4%
9	35	50% × 100%	50%	50%	90%	= 50% × 90% = 45%

For meaningful grouping of salicylates (hazard characterization of salicylic acid), several aspects must be considered, namely bioavailability, metabolism and elimination. Regarding bioavailability, chemicals with comparable physicochemical properties (e.g., MW, LogP and VP) should be assessed together. The substance should be shown to be metabolized to the common metabolite (salicylic acid). Finally, the elimination should be similar within a group, with substances exhibiting similar toxicokinetic profiles reflecting the amount of salicylic acid generated over 24 h. When developing a fit-for-purpose approach for chemical risk management i.e., evaluating hazard and exposure, the chemical similarity, physicochemical properties and toxicokinetic profiles should be considered in addition to similar biological activities (i.e., MoA, toxicodynamics).

In conclusion, grouping of chemicals is intended to help speed up the risk assessment of REACH chemicals. However, grouping should not be based solely on chemical similarity. As mentioned in the guidelines, chemicals should also be grouped according to ADME properties and toxicokinetics profiles relevant to their bioavailability so that differences in internal exposure are considered in the safety assessment. We present here a concept by which salicylates can be grouped according to the extent of systemic exposure to a common metabolite, salicylic acid, after exposure via the dermal route. The results indicate that these chemicals have different physicochemical properties which result in differences in skin absorption and hydrolysis to salicylic acid. Consequently, these different groups of salicylates should be assessed separately.

## Supplementary Information

Below is the link to the electronic supplementary material.

Supplementary file1 (XLSX 24 KB)

Supplementary file2 (XLSX 22 KB)

Supplementary file3 (XLSX 97 KB)

Supplementary file4 (XLSX 21 KB)

## Data Availability

The raw data supporting the conclusions of this article will be made available by the authors, without undue reservation.

## Abbreviations

ADME: Absorption, distribution, metabolism and excretion
ARN: Assessment of regulatory needs
CSS: Chemicals strategy for sustainability
D: Dermis
ECHA: European chemicals agency
EU: European union
HTPK: High-throughput pharmacokinetic
IRS: Integrated regulatory strategy
MF: Metabolism factor
MoA: Mode of action
MW: Molecular weight
OECD: Organization for economic cooperation and development
RF: Receptor fluid
SCCS: Scientific committee on consumer safety
SC: Stratum corneum
VE: Viable epidermis
