# Correction: Grouping of chemicals for safety assessment: the importance of toxicokinetic properties of salicylate esters

**DOI:** 10.1007/s00204-025-04017-z

**Published:** 2025-03-25

**Authors:** Abdulkarim Najjar, Sébastien Grégoire, Beate Nicol, Andreas Natsch, Nazanin Golbamaki, Fanny Boisleve, Amaia Irizar, Brian Wall, Angus Swinscoe, Valérie Masini-Etévé, Kaushal Joshi, Anne Marie Api, Peter Griem, Allison Reis, Nicola J. Hewitt, Estefania Cardamone

**Affiliations:** 1 Beiersdorf AG, Unnastrasse 48, 20245 Hamburg, Germany; 2 L’Oreal Research & Innovation, Aulnay-Sous-Bois, France; 3 Unilever U.K, Colworth Science Park, Sharnbrook, UK; 4 Givaudan Schweiz AG, CH-8600 Duebendorf, Switzerland; 5 Chanel, Neuilly, France; 6 The International Fragrance Association (IFRA), Geneva, Switzerland; 7 Colgate-Palmolive Company, Piscataway, NJ 08854 USA; 8 The Estée Lauder Companies, Whitman Laboratories, Petersfield, UK; 9 Research Institute for Fragrance Materials (RIFM), Inc, Mahwah, NJ USA; 10 Symrise AG, Holzminden, Germany; 11 The Procter & Gamble Company, Mason, OH 45040 USA; 12 Scientific Writing Services, Erzhausen, Germany; 13 Cosmetics Europe, Brussels, Belgium

**Correction: Archives of Toxicology (2025) 99:995–1010** 10.1007/s00204-024-03935-8

In this article, the authors regret that Eq. 10 contained an error. This is used for Eq. 9, which describes the evaporation rate for a pure chemical. However, all evaluated substances were applied as a diluted solution in a vehicle, not as a pure chemical. The time in which the chemical completely disappears from the skin surface by evaporation can be extremely short, e.g. a few seconds, and this process depends on whether the chemical is applied pure or diluted in a vehicle. If the equation for the pure chemical is used for the condition of use (2 mg/cm^2^ of formulation containing chemical at 1%), this can lead to the underestimation of skin absorption. To overcome this constraint, a corrective factor for the dilution effect is introduced in the calculation of the mass transfer coefficient (Eq. 10). Air velocity, u, is normalized to the concentration of chemical in the formula expressed in % (w/w).

Equation 10 and the accompanying text should therefore be follows:

Incorrect Eq. (10)

$${k}_{g}= \frac{{6.320u}^{0.78}}{{MW}^{1/3}}$$

Correct Eq. (10)

$${k}_{g}=\frac{6.320 {\left(\frac{u}{C/100}\right)}^{0.78}}{{MW}^{1/3}},$$

where C is the concentration of chemical (expressed as a % (w/w)) and *u* is the air velocity (with a value of 0.165 m.s^−1^) adjacent to the skin (Stempfer and Bunge 2005).

